# Supplementary material for: Host and antibiotic jointly select for greater virulence in Staphylococcus aureus
Source: eLife. 2026 Jun 16;14:RP107936. doi: 10.7554/eLife.107936 (PMC13271738; doi:10.7554/eLife.107936)
Supplement: Supplementary file 1. [file elife-107936-supp1.docx]

| **Gene** | **Role identified in previous studies** | **Number of mutations in our study** | **Notes** |
| --- | --- | --- | --- |
| *agr* | regulator, virulence (Traber et al., 2008) | 22 | 14 in MRSA  18 in -OX  10 in +HOST |
| *saeRS* | regulator, virulence (Liang et al., 2006) | 4 | 3 in MRSA |
| *codY* | regulator, metabolism and virulence (Majerczyk et al., 2008) | 8 | 6 in MSSA  8 in +HOST  7 in +OX |
| *graSR* | regulator, virulence (Falord et al., 2011) and resistance to beta-lactam antibiotics (Müller et al., 2018) | 4 | 4 in MRSA  4 in +OX |
| *pbpA* | resistance to beta-lactam antibiotics (Berti et al., 2016) | 4 | 2 in MRSA  2 MSSA  4 in +OX |
| *pbpB* | resistance to beta-lactam antibiotics (Łȩski and Tomasz, 2005) | 4 | 4 MRSA  4 +OX |
| *gdpP* | resistance to beta-lactam antibiotics (Sommer et al., 2021) | 4 | 1 in MRSA  3 in MSSA  4 in +OX |
| SCC*mec and* Arginine Catabolic Mobile Element (ACME) | resistance to beta-lactam antibiotics (Shore and Coleman, 2013) | 9 | 5 SCC*mec* (23.7 kb deletion)  4 also included ACME (54.7 kb deletion)  6 in MSSA  7 in +HOST  1 *arcA* (99 bp deletion, encoded by ACME) in MRSA |
| *brnQ1* | metabolism (Kaiser et al., 2015) | 20 | 16 in -HOST |
| *purr* | virulence (Goncheva et al., 2020) | 4 | 4 in +HOST  3 in MRSA  3 in +OX |
| *gpmA* | virulence (Radin et al., 2019) | 2 | 2 in +HOST  2 in MSSA  2 in -OX |

**References**

1. Traber KE, Lee E, Benson S, Corrigan R, Cantera M, Shopsin B, Novick RP. 2008. agr function in clinical Staphylococcus aureus isolates. Microbiology 154:2265–2274. DOI: https://doi.org/10.1099/mic.0.2007/ 011874-0, PMID: 18667559
2. Liang X, Yu C, Sun J, Liu H, Landwehr C, Holmes D, Ji Y. 2006. Inactivation of a two-component signal transduction system, SaeRS, eliminates adherence and attenuates virulence of Staphylococcus aureus. Infection and Immunity 74:4655–4665. DOI: https://doi.org/10.1128/IAI.00322-06, PMID: 16861653
3. Majerczyk CD, Sadykov MR, Luong TT, Lee C, Somerville GA, Sonenshein AL. 2008. Staphylococcus aureus CodY negatively regulates virulence gene expression. Journal of Bacteriology 190:2257–2265. DOI: https:// doi.org/10.1128/JB.01545-07, PMID: 18156263
4. Falord M, Mäder U, Hiron A, Débarbouillé M, Msadek T. 2011. Investigation of the Staphylococcus aureus GraSR regulon reveals novel links to virulence, stress response and cell wall signal transduction pathways. PLOS ONE 6:e21323. DOI: https://doi.org/10.1371/journal.pone.0021323, PMID: 21765893
5. Müller A, Grein F, Otto A, Gries K, Orlov D, Zarubaev V, Girard M, Sher X, Shamova O, Roemer T, François P, Becher D, Schneider T, Sahl HG. 2018. Differential daptomycin resistance development in Staphylococcus aureus strains with active and mutated gra regulatory systems. International Journal of Medical Microbiology 308:335–348. DOI: https://doi.org/10.1016/j.ijmm.2017.12.002, PMID: 29429584
6. Berti AD, Theisen E, Sauer JD, Nonejuie P, Olson J, Pogliano J, Sakoulas G, Nizet V, Proctor RA, Rose WE. 2016. Penicillin binding protein 1 is important in the compensatory response of Staphylococcus aureus to daptomycin-induced membrane damage and is a potential target for β-lactam–daptomycin synergy. Antimicrobial Agents and Chemotherapy 60:451–458. DOI: https://doi.org/10.1128/AAC.02071-15, PMID: 26525797
7. Leski TA, Tomasz A. 2005. Role of penicillin-binding protein 2 (PBP2) in the antibiotic susceptibility and cell wall cross-linking of Staphylococcus aureus: evidence for the cooperative functioning of PBP2, PBP4, and PBP2A. Journal of Bacteriology 187:1815–1824. DOI: https://doi.org/10.1128/JB.187.5.1815-1824.2005, PMID: 15716453
8. Sommer A, Fuchs S, Layer F, Schaudinn C, Weber RE, Richard H, Erdmann MB, Laue M, Schuster CF, Werner G, Strommenger B. 2021. Mutations in the gdpP gene are a clinically relevant mechanism for β-lactam resistance in meticillin-resistant Staphylococcus aureus lacking mec determinants. Microbial Genomics 7:1–13. DOI: https://doi.org/10.1099/mgen.0.000623, PMID: 34486969
9. Shore AC, Coleman DC. 2013. Staphylococcal cassette chromosome mec: recent advances and new insights. International Journal of Medical Microbiology 303:350–359. DOI: https://doi.org/10.1016/j.ijmm.2013.02.002, PMID: 23499303
10. Kaiser JC, Omer S, Sheldon JR, Welch I, Heinrichs DE. 2015. Role of BrnQ1 and BrnQ2 in branched-chain amino acid transport and virulence in Staphylococcus aureus. Infection and Immunity 83:1019–1029. DOI: https://doi. org/10.1128/IAI.02542-14, PMID: 25547798
11. Goncheva MI, Flannagan RS, Heinrichs DE. 2020. De Novo purine biosynthesis is required for intracellular growth of Staphylococcus aureus and for the hypervirulence phenotype of a purR Mutant. Infection and Immunity 88:e00104-20. DOI: https://doi.org/10.1128/IAI.00104-20, PMID: 32094249
12. Radin JN, Kelliher JL, Solórzano PKP, Grim KP, Ramezanifard R, Slauch JM, Kehl-Fie TE. 2019. Metal-independent variants of phosphoglycerate mutase promote resistance to nutritional immunity and retention of glycolysis during infection. PLOS Pathogens 15:e1007971. DOI: https://doi.org/10.1371/journal.ppat.1007971, PMID: 31344131
