# Supplementary material for: Host and antibiotic jointly select for greater virulence in Staphylococcus aureus
Source: eLife. 2026 Jun 16;14:RP107936. doi: 10.7554/eLife.107936 (PMC13271738; doi:10.7554/eLife.107936)
Supplement: Supplementary file 2. — SYN = synonymous, NONSYN = nonsynonymous. [file elife-107936-supp2.docx]

| **Gene** | **Evolution treatment** | **Population** | **Position** | **Ancestral codon** | **Evolved codon** | **Mutation type** | **Mutation category** | **Amino acid change** |
| --- | --- | --- | --- | --- | --- | --- | --- | --- |
| ACME and *SCCmec* | MRSA -host -ox | three | 33633 | 54659-bp |  | DEL | indel |  |
|  | MSSA +host +ox | three | 33633 | 54659-bp |  | DEL | indel |  |
|  | MSSA +host -ox | five | 33633 | 54659-bp |  | DEL | indel |  |
|  |  | six | 33633 | 54659-bp |  | DEL | indel |  |
| B7H15_10520 | MRSA +host +ox | six | 2031543 | T | C | SNP | SYN | I19I |
|  | MRSA -host +ox | three | 2031543 | T | C | SNP | SYN | I19I |
| *SCCmec* | MRSA +host -ox | three | 33636 | 23687-bp |  | DEL | indel |  |
|  |  | six | 33636 | 23687-bp |  | DEL | indel |  |
|  | MSSA +host -ox | four | 33636 | 23687-bp |  | DEL | indel |  |
|  |  | five | 33636 | 23687-bp |  | DEL | indel |  |
|  | MSSA -host +ox | two | 33636 | 23687-bp |  | DEL | indel |  |
| acetyl-CoA synthetase/  antibiotic biosynthesis monooxy-genase | MRSA +host -ox | two | 2749064 | C | A | SNP | intergenic |  |
|  | MSSA +host -ox | one | 2749081 | T | A | SNP | intergenic |  |
| *agr* | MRSA +host +ox | six | 2151051 | C | T | SNP | NONSYN | S164L |
|  |  | six | 2151048 | A | G | SNP | NONSYN | E163G |
|  | MRSA +host -ox | one | 2151213 | G | T | SNP | NONSYN | R218L |
|  |  | two | 2150580 | A | C | SNP | NONSYN | E7A |
|  |  | four | 2150063 | A | T | SNP | other | K256* |
|  | MRSA +host -ox | five | 2151272 | A |  | DEL | indel |  |
|  |  | six | 2150931 | T |  | DEL | indel |  |
|  | MRSA -host +ox | three | 2151051 | C | T | SNP | NONSYN | S164L |
|  |  | three | 2151048 | A | G | SNP | NONSYN | E163G |
|  | MRSA -host -ox | two | 2149664 | A |  | DEL | indel |  |
|  |  | three | 2151047 | G | T | SNP | other | E163* |
|  |  | four | 2151272 | A |  | DEL | indel |  |
|  |  | five | 2149476 | G | A | SNP | other | W60* |
|  |  | six | 2151212 | C | T | SNP | other | R218* |
|  | MSSA +host -ox | four | 2150250 | G | A | SNP | NONSYN | G318D |
|  |  | five | 2151233 | A | T | SNP | other | K225* |
|  |  | six | 2150298 | C | A | SNP | NONSYN | P334H |
|  | MSSA -host -ox | one | 2149635 | C | A | SNP | other | S113* |
|  |  | three | 2150803 | T | CAGTT | INS | indel |  |
|  |  | four | 2149989 | C | A | SNP | NONSYN | T231K |
|  |  | six | 2150700 | C | A | SNP | NONSYN | A47D |
|  |  | six | 2150691 | TT | AA | SUB | indel |  |
| *agrD/C* | MRSA -host +ox | one | 2149252 | G | A | SNP | intergenic |  |
|  | MSSA -host -ox | two | 2149281 | T | G | SNP | intergenic |  |
| alr | MRSA -host +ox | two | 2190276 | A | G | SNP | NONSYN | L280S |
|  |  | six | 2190276 | A | G | SNP | NONSYN | L280S |
|  | MRSA -host -ox | one | 2190276 | A | G | SNP | NONSYN | L280S |
| *argR* | MRSA +host +ox | four | 1639665 | G | C | SNP | NONSYN | T99R |
|  | MRSA -host +ox | two | 1639510 | A | T | SNP | NONSYN | *151K |
|  |  | six | 1639510 | A | T | SNP | NONSYN | *151K |
|  | MRSA -host -ox | one | 1639510 | A | T | SNP | NONSYN | *151K |
| arginine repressor/-geranyl transferase | MRSA +host -ox | three | 1639991 | C | T | SNP | intergenic |  |
|  | MSSA +host -ox | four | 1639991 | C | T | SNP | intergenic |  |
|  |  | six | 1639991 | C | T | SNP | intergenic |  |
| *brnQ1* | MRSA -host -ox | two | 218284 | A | G | SNP | NONSYN | L154S |
|  |  | three | 218456 | A | T | SNP | NONSYN | F97I |
|  |  | three | 218451 | G |  | DEL | indel |  |
|  |  | four | 218425 | 108-bp |  | DEL | indel |  |
|  |  | five | 217434 | GCCGATAATGAA |  | DEL | indel |  |
|  |  | six | 217538 | 97-bp |  | DEL | indel |  |
|  | MSSA +host +ox | three | 218420 | CA | AT | SUB | indel |  |
|  | MSSA +host -ox | two | 218581 | A | T | SNP | other | L55* |
|  |  | three | 217879 | G | T | SNP | other | S289* |
|  |  | five | 218644 | C | A | SNP | NONSYN | G34V |
|  | MSSA -host +ox | one | 218035 | C | T | SNP | NONSYN | G237D |
|  |  | two | 218420 | CA | AT | SUB | indel |  |
|  |  | three | 218420 | CA | AT | SUB | indel |  |
|  |  | four | 218035 | C | T | SNP | NONSYN | G237D |
|  |  | five | 218420 | CA | AT | SUB | indel |  |
|  |  | six | 218035 | C | T | SNP | NONSYN | G237D |
|  | MSSA -host -ox | one | 218128 | GCTAGCGCAT |  | DEL | indel |  |
|  |  | two | 218644 | C | A | SNP | NONSYN | G34V |
|  |  | three | 218644 | C | A | SNP | NONSYN | G34V |
|  |  | six | 218644 | C | A | SNP | NONSYN | G34V |
| *codY* | MRSA +host +ox | two | 1275011 | C | T | SNP | NONSYN | H159Y |
|  |  | six | 1274675 | A | G | SNP | NONSYN | K47E |
|  |  | one | 1275155 | G | T | SNP | NONSYN | A207S |
|  |  | two | 1275155 | G | T | SNP | NONSYN | A207S |
|  | MSSA +host +ox | four | 1275155 | G | T | SNP | NONSYN | A207S |
|  |  | five | 1275155 | G | T | SNP | NONSYN | A207S |
|  |  | six | 1275155 | G | T | SNP | NONSYN | A207S |
|  | MSSA +host -ox | two | 1274976 | C | T | SNP | NONSYN | A147V |
| copper-translo-cating P-type ATPase | MRSA +host +ox | six | 87329 | T | A | SNP | SYN | L624L |
|  | MRSA -host +ox | three | 87329 | T | A | SNP | SYN | L624L |
| *ebh* | MSSA +host -ox | five | 1477894 | C | T | SNP | NONSYN | E8357K |
|  | MSSA -host -ox | two | 1480506 | G | A | SNP | NONSYN | P7486L |
| *fmtB* | MRSA +host +ox | six | 2284788 | G | T | SNP | other |  |
|  | MRSA -host +ox | three | 2284788 | G | T | SNP | other |  |
| *gdpP* | MRSA +host +ox | three | 18721 | G | T | SNP | NONSYN | G307V |
|  | MSSA +host +ox | one | 18720 | G | A | SNP | NONSYN | G307S |
|  |  | four | 18745 | A | C | SNP | NONSYN | K315T |
|  |  | five | 19143 | A | T | SNP | NONSYN | S448C |
| *gpmA* | MSSA +host -ox | one | 2539086 | G | A | SNP | NONSYN | R114C |
|  | MSSA +host -ox | three | 2538887 | G | A | SNP | NONSYN | S180F |
| *graSR* | MRSA +host +ox | three | 734510 | A |  | DEL | indel |  |
|  |  | four | 735131 | C | G | SNP | other | S187* |
|  |  | six | 734085 | T |  | DEL | indel |  |
|  | MRSA -host +ox | three | 734085 | T |  | DEL | indel |  |
| Hydroxy-methyl-glutaryl-CoA synthase/-cysteine methyl-transferase | MRSA +host +ox | six | 2685330 | T | C | SNP | intergenic |  |
|  | MRSA -host +ox | three | 2685330 | T | C | SNP | intergenic |  |
| *pbpA* | MRSA +host +ox | one | 1189756 | C | A | SNP | NONSYN | D350E |
|  |  | four | 1190201 | C | G | SNP | NONSYN | H499D |
|  | MSSA +host +ox | three | 1189756 | C | A | SNP | NONSYN | D350E |
|  |  | six | 1189758 | G | T | SNP | NONSYN | W351L |
| *pbpB* | MRSA +host +ox | two | 1520209 | G | A | SNP | NONSYN | A132T |
|  |  | five | 1520434 | G | A | SNP | NONSYN | A207T |
|  | MRSA -host +ox | one | 1520247 | A | GGTTCTGAA | INS | indel |  |
|  |  | four | 1520414 | G | T | SNP | NONSYN | G200V |
| *purR* | MRSA +host +ox | one | 532212 | C | T | SNP | NONSYN | R96C |
|  |  | three | 532013 | T | T | INS | indel |  |
|  |  | five | 532013 | T | T | INS | indel |  |
|  | MRSA -host +ox | four | 532240 | T | T | INS | indel |  |
| *saeRS* | MRSA +host +ox | two | 782595 | G | T | SNP | NONSYN | P74T |
|  |  | five | 782577 | C | T | SNP | NONSYN | A80T |
|  | MRSA +host -ox | two | 781408 | G | A | SNP | NONSYN | R241C |
|  | MSSA +host +ox | six | 782211 | C |  | DEL | indel |  |
