# Supplementary material for: Host and antibiotic jointly select for greater virulence in Staphylococcus aureus
Source: eLife. 2026 Jun 16;14:RP107936. doi: 10.7554/eLife.107936 (PMC13271738; doi:10.7554/eLife.107936)
Supplement: Supplementary file 3. — All degrees of freedom equaled 1. [file elife-107936-supp3.docx]

| **treatment** | **gene** | **chi-square** | **P-value** |
| --- | --- | --- | --- |
| MRSA +HOST +OX | *pbpA* | 21.626 | < 0.001 |
| MSSA +HOST +OX | *pbpA* | 26.431 | < 0.001 |
| MRSA +HOST +OX | *saeRS* | 4.8088 | 0.02832 |
| MRSA +HOST +OX | *purR* | 4.3463 | 0.03709 |
| MRSA +HOST +OX | *pbpb* | 11 | 0.000911 |
| MRSA -HOST +OX | *pbpb* | 8.2065 | 0.004174 |
| MRSA -HOST -OX | *brnQ1* | 27.748 | < 0.001 |
| MSSA +HOST -OX | *brnQ1* | 27.748 | < 0.001 |
| MSSA -HOST -OX | *brnQ1* | 19.773 | < 0.001 |
| MSSA -HOST +OX | *brnQ1* | 23.097 | < 0.001 |
| MRSA +HOST +OX | *codY* | 0.19574 | 0.6582 |
| MSSA +HOST -OX | *codY* | 1.6584 | 0.2914 |
| MRSA +HOST +OX | *graSR* | 0.19574 | 0.6582 |
| MRSA +HOST +OX | *gdpP* | 2.8047 | 0.09399 |
| MRSA +HOST +OX | *agr* | 0.3538 | 0.552 |
| MRSA +HOST -OX | *agr* | 0.35066 | 0.5537 |
| MRSA -HOST +OX | *agr* | 0.3538 | 0.552 |
| MRSA -HOST -OX | *agr* | 0.223 | 0.6368 |
| MSSA +HOST -OX | *agr* | 0.34068 |  |
| MSSA -HOST -OX | *agr* | 0.2662 | 0.6059 |
| MRSA -HOST -OX | *argR* | 0.223 | 0.6368 |
| MRSA -HOST +OX | *argR* | 0.3538 | 0.552 |
