# Supplementary material for: Host and antibiotic jointly select for greater virulence in Staphylococcus aureus
Source: eLife. 2026 Jun 16;14:RP107936. doi: 10.7554/eLife.107936 (PMC13271738; doi:10.7554/eLife.107936)
Supplement: Supplementary file 4. [file elife-107936-supp4.docx]

| **Category** | **“isolation_source” term** |
| --- | --- |
| blood/systemic | blood  bacteremia  csf  bronchial  aortic  osteomyelitis  respiratory  sputum  bronch  septicaemia  pericar  kidney  liver  spleen  brain  bone  lung  spine  spinal  periton  pancreas  joint  trachea  urethra  uterus  airway fluid  bile  ascitic fluid  bsi  bal  bone marrow  broncoscopy  bronscoscopy  cerebrospinal fluid  endotracheal aspirate  endotrracheal secretion  trachael aspirate  expectoration  hematoma  pleural fluid  septic arthritis  skeletal system  synovial fluid  thoracic cavity  thymic pleural effusion  tissue - aortic valve  yolk sac infection  urinary tract infection  arthritis aspirates  necrotizing  “wound culture from necrotizing fasciitis patient” |
| skin/nose/throat | abcess  abscess  asbcess  absess  nare  naso  sinus  nasal  nostril  cellulitis  wound  burn  sore  pus  ulcer  lesion  swab  screen  abdom  buttock  eye  groin  leg  arm  back  oral  hand  cheek  chest  elbow  foot  femur  axilla  knee  neck  ankle  thigh  cornea  graft  umbillicus  umbilicus  breast  ear  ulcus cruris  darcocystitis  decubitus ulcer  empyema  face  fluid breast  granuloma  hip - left  hip infection  inguinal  left hip aspiration  oropharynx  palm  penis  perineal  perineum  periodontal  rectum  sample from soft tissue  soft tissue  scapula  secretion left hip  ssti |
| general host-association | biological sample  biological liquide  aspirate  aspiration  biopsy  excreted bodily substance  tissue  milk  urine  bodily fluid  vaginal  feces  stool  tissues  body fluid  faecal  infection  infection site  mass  post surgical secretion  secretion surgical  surgical site infection  chronic  colonisation  colonization  commensal  community aquired  clinical  human  patient  male  hcw  host |
| animal | chimpanzee  cloudrat  corncrake  cow  sheep  macaque  lion  bat  chicken  pork  horse  canine  pig  poultry  meat  capybara  chaffinch  meerkat  mongoose  monkey  parrot  pheasant  porpoise  rabbit  seal  sparrow  tapir  wildbird  turkey  cat  goat  great tit  ground beef  ground turkey  guineapig  claw  paw  tail  diced chiken  feline colonisation  “rat feces from intestine (rattus norvegicus)”  veal calf  veterinary/diagnostic sample  non-migratory seabirds |
| environment | various material  cryotube from air metagenome  fish drying yard  glove  gown  hardware  heart valve  hexachlorocyclohexane-contaminated soil  household surface  jp drainage  catheter  minibal  pacemaker wire  peg tube drainage  peri-bypass anterior material  pin tract  surface veterinary clinic  surgical ward  suture |
| others | culture  lab  physical  food  facility  acute  atcc  isolate  hospital  in vitro derived  in vitro evolution  blops  case  drain  drainage  fluid  icu  index  mara  mrsa broth  non-icu  norway  ny  clinic  pool  pooled  presumed outlier  serial passagee experiment  staphylococcus aureus usa300  surface  surgical site  tip |
| missing | not applicable  not collected  not known  unknown  NA  missing  not avail  unknonwn  unknow  unspecified |
