# Supplementary material for: Host and antibiotic jointly select for greater virulence in Staphylococcus aureus
Source: eLife. 2026 Jun 16;14:RP107936. doi: 10.7554/eLife.107936 (PMC13271738; doi:10.7554/eLife.107936)
Supplement: Peer review history

# Editor report: eLife Assessment

**DOI:** 10.7554/eLife.107936.3.sa0

**Authors:** Marianoel Pereira-Gómez

**Affiliations:** Universidad de la República Uruguay

**Keywords:** Convincing, Important

This **important** study examines the evolution of virulence and antibiotic resistance in *Staphylococcus aureus* under multiple selection pressures, specifically host immune function and antibiotic exposure. The evidence presented is **convincing**, supported by rigorous phenotypic and genomic data from within-host evolution experiments. The manuscript now provides a nuanced and robust interpretation of how pathogens adapt to complex selective landscapes.

# Referee report: Reviewer #1 (Public review)

**DOI:** 10.7554/eLife.107936.3.sa1

Summary:

The authors investigate how methicillin-resistant (MRSA) and sensitive (MSSA) *Staphylococcus aureus* adapt to a new host (*C. elegans*) in the presence or absence of a low dose of the antibiotic oxacillin. Using an "Evolve and Resequence" design with 48 independently evolving populations, they track changes in virulence, antibiotic resistance, and other fitness-related traits over 12 passages. Their key finding is that selection from both the host and the antibiotic together, rather than either pressure alone, synergistically results in the evolution of the most virulent pathogens. Genomically, they find that this adaptation repeatedly involves parallel mutations in a small number of key regulatory genes, most notably codY, agr, and saeRS.

Strengths:

The main advantage of the research lies in its strong and thoroughly replicated experimental framework, enabling significant conclusions to be drawn based on the concept of parallel evolution. The study successfully integrates various phenotypic assays (virulence, growth, hemolysis, biofilm formation) with whole-genome sequencing, offering an extensive perspective on the adaptive landscape. The identification of certain regulatory genes as common targets of selection across distinct lineages is an important result that indicates a level of predictability in how pathogens adapt. Furthermore, the detailed mapping of specific parallel mutations provides a highly useful genomic resource for the microbiology community.

Revisions and Re-Appraisal:

In the initial version of the manuscript, a primary limitation was the use of causal language to link specific mutations to phenotypes, despite the evidence from the evolution experiment being correlational. In this revised version, the authors have excellently addressed this limitation. They have meticulously revised the text to accurately reflect these relationships as strong, statistically significant genetic associations rather than confirmed facts. Furthermore, they explicitly acknowledge that future ancestral reconstruction experiments will be required to confirm direct causality. The authors have also appropriately clarified the visual interpretations of their data (such as the PCA clustering) and refined their discussion of mutation rates. With these revisions, the claims made are fully supported by the data presented.

Impact and Context:

The authors successfully achieve their aims, demonstrating that the combined effects of host and antibiotic pressures collaboratively propel the evolution of heightened virulence. While the nematode model does not perfectly mimic human or mammalian infection, the evolutionary principles uncovered here are highly relevant to both evolutionary biology and infectious disease management. The evidence presented is compelling, and the strong correlational hypotheses generated by this study offer a robust and significant basis for upcoming mechanistic research into pathogen adaptation.

Comments on revisions:

I commend the authors for their thorough, thoughtful, and highly constructive revision. You have successfully addressed all of my major and minor comments. The addition of Table S2 and the careful revisions to the causal language have significantly strengthened the manuscript and clarified the data interpretation. I have no further recommendations. Great work!

# Referee report: Reviewer #2 (Public review)

**DOI:** 10.7554/eLife.107936.3.sa2

Summary:

The manuscript describes the results of an evolution experiment where *Staphylococcus aureus* was experimentally evolved via sequential exposure to an antibiotic followed by passaging through *C. elegans* hosts. Because infecting *C. elegans* via ingestion results in lysis of gut cells and an immune response upon infection, the *S. aureus* were exposed separately across generations to antibiotic stress and host immune stress. Interestingly, the dual selection pressure of antibiotic exposure and adaptation to a nematode host resulted in increased virulence of *S. aureus* towards *C. elegans*.

Strengths:

The data presented provide strong evidence that in *S. aureus* traits involved in adaptation to a novel host and those involved in antibiotic resistance evolution are not traded-off. On the contrary, they seem to be correlated, with strains adapted to antibiotics having higher virulence towards the novel host. As increased virulence is also associated with higher rates of haemolysis, these virulence increases are likely to reflect virulence levels in vertebrate hosts.

Weaknesses:

Right now, the results are presented in the context of human infections being treated with antibiotics, which, in my opinion, is inappropriate. This is because

(1) exposure to the host and antibiotics was sequential, not simultaneous, and thus does not reflect the treatment of infection, and

(2) because the site of infection is different in *C. elegans* and human hosts.

Nevertheless, the results are of interest; I just think the interpretation and framing should be adjusted.

Comments on revisions:

Following the revision, I now think the weakness I initially described has been addressed well by the authors.

# Referee report: Reviewer #3 (Public review)

**DOI:** 10.7554/eLife.107936.3.sa3

Summary:

Su et al. sought to understand how the opportunistic pathogen *Staphylococcus aureus* responds to multiple selection pressures during infection. Specifically, the authors were interested in how the host environment and antibiotic exposure impact the evolution of both virulence and antibiotic resistance in *S. aureus*. To accomplish this, the authors performed an evolution experiment where *S. aureus* were fed to *Caenorhabditis elegans* as a model system to study the host environment and then either subjected to the antibiotic oxacillin or not. Additionally, the authors investigated the difference in evolution between an antibiotic-resistant stain MRSA and an isogenic susceptible strain MSSA. They found that MRSA strains evolved in both antibiotic and host conditions became more virulent and that strains evolved outside these conditions lost virulence. Looking at the strains evolved in just antibiotic conditions, the authors found that *S. aureus* maintained its ability to lyse blood cells. Mutations in codY, gdpP and pbpA were found to be associated with increased virulence. Additionally, these mutations identified in these experiments were found in *S. aureus* strains isolated from human infections.

Strengths:

The data are well-presented, thorough, and are an important addition to the understanding of how certain pathogens might adapt to different selective pressures in complex environments.

Comments on revisions:

For the most part, my comments have been addressed. It seems that the authors have not addressed my comments about quantifying population sizes in order to understand mutation supply, particularly in light of which experimental phase exhibits the strongest selection and possible increases in mutation rates. While I think this information would be very useful if they had collected it during the experiment, I don't think it is important enough to require additional experiments. I am therefore satisfied with the current state of the manuscript.

# Author comment: Author response

**DOI:** 10.7554/eLife.107936.3.sa4

**Authors:** Michelle Su, Kim Hoang, McKenna Penley, Michelle Davis, Jennifer Gresham, Levi Morran, Timothy Read

**Affiliations:** Emory University Atlanta United States; Emory University Atlanta United States; Emory University Atlanta United States; Emory University Atlanta United States; Emory University Atlanta United States; Emory University Atlanta United States; Emory University Atlanta United States

The following is the authors’ response to the original reviews.

**eLife Assessment**

This important study examines the evolution of virulence and antibiotic resistance in *Staphylococcus aureus* under multiple selection pressures. The evidence presented is convincing, with rigorous data that characterizes the outcomes of the evolution experiments. However, the manuscript's primary weakness is in its presentation, as claims about the causal relationship between genotypes and phenotypes are based on correlational evidence. The manuscript needs to be revised to address these limitations, clarify the implications of the experimental design, and adjust the overall narrative to better reflect the nature of the findings.

Thank you for your feedback. Here, we summarize the major changes made in the revised manuscript:

(1) We did not test causality between mutations and phenotypes in our study. We were intentional about not using causal wording (“mutation X caused/led to/resulted in phenotype Y”), and only discussed these results using the terms “correlation” and “association”, and only when they were statistically significant. We understand that some readers may view these terms as being equivalent to “causation”, thus in the revision, we have modified our wording as suggested (please see below for specific lines).

(2) We agree that experimental evolution in nematodes is not a direct simulation of evolution in humans. The goal of our study was first and foremost, a test of how multiple selective pressures can shape pathogen evolution. This point was presented in the first paragraph, the second to last paragraph of the Introduction (which included our hypotheses), and the last paragraph of the manuscript. References to humans and other mammalian systems were intended to point out similarities between our findings and what had already been found in *S. aureus* outside the lab. Despite differences between mammals and nematodes, several parallels arose at both the phenotypic and genomic levels, which is interesting from an evolutionary standpoint. We understand that more experiments and tests would be needed before we can make claims about the selective pressures acting on *S. aureus* outside the lab. We presented some information in the context of humans because a large part of the literature on *S. aureus* is on its role as a major bacterial pathogen; we did not want to neglect this aspect of its natural life history.

In the revised manuscript, we are more explicit in stating these points, as well as tempering some language regarding human infection, and removing some references to humans. Please see below for specific lines as well as justification for specific references to humans/mammalian systems.

(3) We have including additional details on the experimental design below. We hope this is sufficiently clarifying.

**Public Reviews:**

**Reviewer #1 (Public review):**

Summary:

The authors investigate how methicillin-resistant (MRSA) and sensitive (MSSA) *Staphylococcus aureus* adapt to a new host (*C. elegans*) in the presence or absence of a low dose of the antibiotic oxacillin. Using an "Evolve and Resequence" design with 48 independently evolving populations, they track changes in virulence, antibiotic resistance, and other fitness-related traits over 12 passages. Their key finding is that selection from both the host and the antibiotic together, rather than either pressure alone, results in the evolution of the most virulent pathogens. Genomically, they find that this adaptation repeatedly involves mutations in a small number of key regulatory genes, most notably codY, agr, and saeRS.

Strengths:

The main advantage of the research lies in its strong and thoroughly replicated experimental framework, enabling significant conclusions to be drawn based on the concept of parallel evolution. The study successfully integrates various phenotypic assays (virulence, growth, hemolysis, biofilm formation) with whole-genome sequencing, offering an extensive perspective on the adaptive landscape. The identification of certain regulatory genes as common targets of selection across distinct lineages is an important result that indicates a level of predictability in how pathogens adapt.

Thank you very much.

Weaknesses:

(1) The main limitation of the paper is that its findings on the function of specific genes are based on correlation, not cause-and-effect evidence. While the parallel evolution evidence is strong, the authors have not yet performed the definitive tests (i.e., reconstruction of ancestral genes) to ensure that the mutations identified in isolation are enough to account for the virulence or resistance changes observed. This makes the conclusions more like firm hypotheses, not confirmed facts.

We have replaced instances of “association” and “correlation” with wording similar to that suggested where applicable, including:

L 342 – 344: “The loss of *SCCmec* and ACME was more often identified in populations exhibiting an increase in total growth from the ancestor outside the host…”

L 371 – 375: “Mutations in three genes were regularly identified in populations exhibiting significant increases in virulence from the ancestor: *codY*, *gdpP*, and *pbpA*. Mutations in *agr* in general were not associated with changes in overall virulence, but MSSA populations harboring mutations in this gene were more likely to exhibit greater virulence compared to MRSA populations (Wilcoxon rank sum exact test P = 0.045).”

L 377: “Mutations in specific genes were often found in populations able to hemolyze red blood cells…”

L 379 – 381: “There were also significant differences between the mutations regularly identified in oxacillin-resistant populations evolved from the MSSA ancestor...”

L 384 – 385: “By contrast, mutations in *agr* were often in populations exhibiting loss of hemolytic activity, consistent with previous findings...”

L 409 – 410: “Mutations that arose during experimental evolution are regularly found in strains associated with human systemic infections.”

We have also stated that ancestral reconstruction is needed:

L 553 – 555: “Future experiments may include introducing these mutations into the ancestral background to directly link the mutations in these genes to evolved virulence.”

(2) In some instances, the claims in the text are not fully supported by the visual data from the figures or are reported with vagueness. For example, the display of phenotypic clusters in the PCA (Figure 6A) and the sweeping generalization about the effect of antibiotics on the mutation rates (Figure S5) can be more precise and nuanced. Such small deviations dilute the overall argument somewhat and must be corrected.

In reference to Fig. 6A, we have revised the statement as suggested: “…where populations exposed to host and sub-MIC oxacillin clustered together, largely separating from all other treatments…” Line 442

In reference to Fig. S5, we conducted statistics to include both MRSA and MSSA populations and examined the effect of oxacillin on the number of mutations. While oxacillin had a significant effect on the number of mutations, we agree with the reviewer that this may be driven by the MRSA populations and have clarified: “Sub-MIC oxacillin selection also resulted in more mutations than in its absence ($\chi_{1}^{2}$ = 5.92, P = 0.015), although this is likely driven by MRSA populations.” Lines 310 – 311

**Reviewer #2 (Public review):**

Summary:

The manuscript describes the results of an evolution experiment where *Staphylococcus aureus* was experimentally evolved via sequential exposure to an antibiotic followed by passaging through *C. elegans* hosts. Because infecting *C. elegans* via ingestion results in lysis of gut cells and an immune response upon infection, the *S. aureus* were exposed separately across generations to antibiotic stress and host immune stress. Interestingly, the dual selection pressure of antibiotic exposure and adaptation to a nematode host resulted in increased virulence of *S. aureus* towards *C. elegans*.

Strengths:

The data presented provide strong evidence that in *S. aureus*, traits involved in adaptation to a novel host and those involved in antibiotic resistance evolution are not traded off. On the contrary, they seem to be correlated, with strains adapted to antibiotics having higher virulence towards the novel host. As increased virulence is also associated with higher rates of haemolysis, these virulence increases are likely to reflect virulence levels in vertebrate hosts.

Weaknesses:

Right now, the results are presented in the context of human infections being treated with antibiotics, which, in my opinion, is inappropriate. This is because

(1) exposure to the host and antibiotics was sequential, not simultaneous, and thus does not reflect the treatment of infection, and

(2) because the site of infection is different in *C. elegans* and human hosts.

We have removed the two sentences referencing site of infection:

Introduction: “In the host, antibiotic concentrations will gradually decline after administration due to metabolism and excretion.”

Discussion: “…in addition to infection of antibiotic-treated hosts, where there is uneven distribution of drugs across tissues.”

For our rationale for discussing humans in general, please see below.

Nevertheless, the results are of interest; I just think the interpretation and framing should be adjusted.

Thank you very much.

**Reviewer #3 (Public review):**

Summary:

Su et al. sought to understand how the opportunistic pathogen *Staphylococcus aureus* responds to multiple selection pressures during infection. Specifically, the authors were interested in how the host environment and antibiotic exposure impact the evolution of both virulence and antibiotic resistance in *S. aureus*. To accomplish this, the authors performed an evolution experiment where *S. aureus* was fed to *Caenorhabditis elegans* as a model system to study the host environment and then either subjected to the antibiotic oxacillin or not. Additionally, the authors investigated the difference in evolution between an antibiotic-resistant strain, MRSA, and an isogenic susceptible strain, MSSA. They found that MRSA strains evolved in both antibiotic and host conditions became more virulent, and that strains evolved outside these conditions lost virulence. Looking at the strains evolved in just antibiotic conditions, the authors found that *S. aureus* maintained its ability to lyse blood cells. Mutations in codY, gdpP, and pbpA were found to be associated with increased virulence. Additionally, these mutations identified in these experiments were found in *S. aureus* strains isolated from human infections.

Strengths:

The data are well-presented, thorough, and are an important addition to the understanding of how certain pathogens might adapt to different selective pressures in complex environments.

Thank you very much.

Weaknesses:

There are a few clarifications that could be made to better understand and contextualize the results. Primarily, when comparing the number of mutations and selection across conditions in an evolution experiment, information about population sizes is important to be able to calculate the mutation supply and number of generations throughout the experiment. These calculations can be difficult in vivo, but since several steps in the methodology require plating and regrowth, those population sizes could be determined. There was also no mention of how the authors controlled the inoculation density of bacteria introduced to each host. This would need to be known to calculate the generation time within the host. These caveats should be addressed in the manuscript.

While the population sizes within hosts and generation time could be determined, we would need to conduct additional experiments (e.g., infecting nematodes with *S. aureus*, then crushing, plating, and counting colony forming units across time intervals) in order to obtain measurements for pathogen growth in hosts across time. For experimental evolution, we crushed a set number of dead nematodes (30) and all bacteria that were released were allowed to grow in liquid media before an aliquot (25%) was used to seed the next passage. Picking and crushing nematodes across 48 populations for one time point was an arduous task. The additional steps of picking, crushing, and plating nematodes across multiple time intervals at the same time experimental evolution was being performed would not be logistically sound.

In terms of the inoculation density of bacteria, all nematodes were placed on abundant lawns of *S. aureus*. Nematodes were exposed to full lawns the entire infection step; bacteria remained in abundance. While we do not know the exact inoculum each individual nematode was exposed to, we know that they ingested the bacteria because of the high mortality rate. Furthermore, we followed the same procedure for every replicate across every host-associated treatment. Host individuals within and across passages were also genetically identical to one another. Altogether, these factors allowed for more consistency across the experiment, such that relative inoculum size should be similar across individual hosts. Please refer to the evolution experiment diagram (Author response image 1) for more details.

Ultimately, while knowing the absolute population size, inoculum size, and generation time within the host is interesting, the rounds of selection (the number of times each population was exposed to the selective pressures) is also important in addressing our major question. Every treatment, which started out from one ancestral clone (MRSA or MSSA), was exposed to the same number of bouts of selection (passages), yet we see significant divergence in terms of traits and mutations. Future directions would certainly involve determining the number of steps (e.g., number of generations within hosts) required to reach these end points, but not knowing exactly how many steps were required do not detract from addressing the larger question of determining how pathogens respond to multiple selective pressures.

Another concern is the number of generations the populations of *S. aureus* spent either with relaxed selection in rich media or under antibiotic pressure in between the host exposure periods. It is probable then that the majority of mutations were selected for in these intervening periods between host infection. Again, a more detailed understanding of population sizes would contribute to the understanding of which phase of the experiment contributed to the mutation profile observed.

We conducted every step of the evolution experiment on the same timeline. For example, all replicates across treatments were grown in liquid media at the same time (see Author response image 1.). All populations were exposed to the same selective pressures at this step of the experiment. We can then compare populations that were subsequently exposed to hosts against those that were not. Populations passaged without a host served as the control. Mutations that were solely unique to host-exposed populations would more likely contribute to the traits of interest, compared to mutations that were in common between the host-exposed and no-host treatments. Similar comparisons could be made with the oxacillin-exposed and no-oxacillin populations.

In general, the only differences between treatments would be driven by the treatments themselves. Given that we are interested in treatment-level effects, any differences in population size or generation time between treatments could contribute to the treatment effects we observe, and thus were not something we aimed to hold uniform across our experiment.

**Author response image 1.** Schematic of procedural steps involved in one passage of *S. aureus* through nematodes (+host -ox) compared to without nematodes (-host -ox).

**Recommendations for the authors:**

**Reviewing Editor Comments:**

We encourage you to address all other comments raised by the reviewers; however, the review team has identified the following points as the most critical and fundamental to improve your manuscript:

(i) Reframing the narrative: You will need to adjust the narrative so that the study is presented as a "proof of principle" rather than a direct simulation of a human infection.

While we referenced human infection, we believe the study had been presented as a proof of principle. Examples include:

(1) We discussed the gap of knowledge in the first paragraph: “It is unclear how virulence evolves in the face of more than one selective pressure and whether this trait is constrained or facilitated by antibiotic resistance.” Lines 86 – 88

(2) In the second to last paragraph in the Introduction, we presented the main hypotheses: “Adaptation may require resources to be expended toward either virulence or antibiotic resistance, leading to a trade-off between these traits (Ferenci, 2016). Alternatively, weaker selection from sub-MIC antibiotics may interact synergistically with hosts and facilitate the evolution or maintenance of high virulence and antibiotic resistance.” Lines 176 – 179

(3) The last paragraph concluded with “Our findings ultimately emphasize the importance of considering the host context in the evolution of antibiotic resistance. Integrating multiple traits, such as virulence, antibiotic resistance, and fitness may be critical in identifying the factors that facilitate host shifts and persistence of drug-resistant pathogens.” Lines 613 – 616

These paragraphs, which set up the context for our work, did not primarily discuss human infections.

In the revised manuscript, we have further tempered language regarding human infection:

L 169 - 172: “Experimentally evolving *S. aureus* in *C. elegans* thus allows us to track the early stages of virulence and antibiotic resistance evolution in novel host populations with the potential to identify conserved genomic regions underlying evolved traits.”

L 595 – 596: “Additional direct tests are needed to evaluate the role of these mutations in adaptation of *S. aureus* to different infection sites.”

L 610 – 611: “Pathogen evolution in a tractable invertebrate animal model yielded phenotypes and genotypes similar to those identified in mammalian hosts, highlighting the utility of evolution experiments to identify potential ecological and genetic mechanisms that may give rise to pathogen traits conserved across systems.”

And removed some references to humans:

In the Introduction: “In the host, antibiotic concentrations will gradually decline after administration due to metabolism and excretion.”

In the Discussion: “…in addition to infection of antibiotic-treated hosts, where there is uneven distribution of drugs across tissues.”

Otherwise, our rationale for referencing humans/mammalian systems in our Introduction include:

Setting the context of our study system: we discussed humans and clinical significance when we first introduced *S. aureus* (lines 132 – 151) and experimental evolution (lines 153 – 172). Much of what is known about *S. aureus* outside the lab is when it is interacting with humans, thus we weaved in relevant information that has been discovered in other organisms.

Hemolysis: This ability is important for *S. aureus* virulence toward *C. elegans* (Sifri et al., 2003).

*S. aureus* genomic database: we intended to leverage this large-scale database of genomes isolated from *S. aureus* outside the lab to compare patterns emerging from experimental evolution to those in existing isolates. Due to its relevance as a major bacterial pathogen, most of the isolates happen to be from clinical settings.

(ii) Adjusting the causal language: You will need to soften the language so that correlational claims do not appear to be causal.

We have adjusted language as noted above.

(iii) Clarifying methodological aspects: You will need to provide more details on the methodology, such as population sizes, and clarify the implications of these in the conclusions of the work.

We have provided additional explanation of methodology and the role of control (no host) treatments above.

**Reviewer #1 (Recommendations for the authors):**

The paper is robust, and the study is of great significance. Tackling the subsequent issues would greatly enhance the paper and elucidate its findings.

Major Recommendations:

(1) Revising Causal Language: The main flaw of the manuscript lies in its presentation of correlational data as if it were causal. We highly suggest a thorough review of the text to soften causal language when connecting genotypes to phenotypes. The absence of ancestral reconstruction should be recognized as a constraint. Assertions ought to be presented as robust, evidence-based hypotheses. For instance, rather than saying a mutation "associated with significant increases in virulence," you might say "was regularly identified in groups that developed increased virulence, strongly suggesting this gene's role in the adaptation." This will more precisely clarify the contribution of the work.

We have softened language and stated that ancestral reconstruction is needed as noted above.

(2) Expand on Parallel Mutations: The examination of parallel evolution in Figure 4A is intriguing but would be notably stronger with additional details. I suggest including an additional supplementary figure or table detailing the specific non-synonymous mutations identified in the highly parallel genes (e.g., codY, agr, gdpP). It is essential for the reader to understand whether parallel evolution is happening at the gene level (different mutations in a single gene) or at the nucleotide level (the precise same mutation appearing again). Kindly specify if any of these mutations were nonsense mutations, as this suggests that the loss-of-function is advantageous.

The full table of mutations is in fig share (10.6084/m9.figshare.28745558). We have added a Supplemental Table (Table S2) containing mutations in genes occurring in more than two populations. Many of these mutations were not the same, indicating parallel evolution at the gene level (lines 315 – 317).

Minor Recommendations for Clarity and Accuracy:

(1) Introduction:

Lines 176-177: Please add a citation for the statement describing the function of the SCCmec cassette, as this is established knowledge.

Done.

(2) Results:

Section Title (Line 254): The title "Host and sub-MIC antibiotic promoted growth..." is imprecise. Figure 3B shows that it is the combination of these factors that promotes growth in MRSA, while oxacillin alone is detrimental. Please revise the title to reflect this synergistic effect.

“Synergistically” has been added to the title: “Host and sub-MIC antibiotic synergistically promoted growth of MRSA…” Lines 269 – 270

Lines 261-263: The description of Figure 3B is incomplete. The text should explicitly state that the -host+ox treatment resulted in the lowest growth for MRSA, which provides a critical contrast and suggests a fitness cost.

We have added “By contrast, exposure to sub-MIC oxacillin alone yielded the lowest growth, suggesting a fitness cost.” Lines 277 – 278

Line 294: The claim that "Sub-MIC oxacillin selection also resulted in more mutations" is a generalization not supported for the MSSA genotype, according to Figure S5. Please revise this sentence to specify that this effect was observed in the MRSA populations.

We have clarified: “Sub-MIC oxacillin selection also resulted in more mutations than in its absence ($\chi_{1}^{2}$ = 5.92, P = 0.015), although this is likely driven by MRSA populations.” Lines 310 – 311

Lines 419-421: The claim that the +host+ox populations in Figure 6A "formed a distinct cluster" is an overstatement, as there is visible overlap with one other treatment (e.g., host-ox). Please revise this to more accurately describe the visual data (e.g., "clustered together, largely separating...").

We have revised the statement as suggested: “…where populations exposed to host and sub-MIC oxacillin clustered together, largely separating from all other treatments…” Lines 442 – 443

Lines 422-424: The interpretation of the MRSA PCA (Figure 6A) focuses on the correlation between virulence and sub-MIC growth. However, the correlation between "biofilm production" and "growth without oxacillin" appears visually stronger. Please address this correlation as well for a more complete interpretation.

We have added “For MRSA populations, biofilm production and growth without oxacillin also appeared to be positively correlated.” Lines 447 – 448

(3) Discussion:

Lines 469-470: The statement that "exposure to oxacillin resulted in pathogens causing the greatest host mortality" is imprecise. The data in Figure 2A show that it is the combination of host and oxacillin. Please revise this for accuracy and add a direct citation to Figure 2A here.

We have added clarification: “Nonetheless, we observed differing evolutionary trajectories, where exposure to oxacillin in host-associated treatments resulted in pathogens causing the greatest host mortality.” Lines 496 – 498

**Reviewer #2 (Recommendations for the authors):**

After reviewing the paper and reading the previous reviews from PLoS Biology, my biggest criticism of the paper is the way the story is told. In principle, the results are interesting and relevant, but the analogy to human infection and immune system/ antibiotic treatment strategies does not fit entirely with the experimental design or the results. I think the motivation needs to be reframed. In the study, antibiotic exposure is purely environmental, i.e., not in the host. How does environmental antibiotic use affect in vivo evolution, as this is not tested? As previous reviewers have pointed out, *S. aureus* is not an enteric pathogen in humans but most often causes skin infections. Furthermore, much of the results and discussion is focused on haemolysis of red blood cells, a cell type that *C. elegans* does not have. What the paper does present, on the other hand, and something that is interesting and novel, is a test in a model system of how a bacterial pathogen evolves to competing selection pressures. I might have hypothesised a priori that these competing pressures result in trade-offs, something which there is no evidence of, even though growth rate does not appear to be negatively impacted as a consequence of selection for drug resistance and virulence together. Instead, many traits are correlated and seemingly at the mechanistic level. This is cool and is a proof of principle, even if the system does not completely mirror reality, and I think the story should be told as such.

We agree entirely with the reviewer that testing how pathogens respond to multiple selective pressures and the resulting lack of trade-offs are significant and interesting. We presented this question (lines 86 – 88) and our hypothesis about such trade-off in the Introduction (lines 176 – 179). As stated above, we had framed our paper to highlight these points and have removed references to antibiotic concentrations in treated humans.

We measured and discussed hemolysis because it is important for virulence toward *C. elegans* (lines 195 – 197) (Sifri et al., 2003). We believe our manuscript contained a reasonable discussion of this trait. For example, three panels of the main figures presented the main hemolysis results (Figures 2B, 2C, and 2D), whereas 23 other panels did not at all involve hemolysis. In the Discussion, hemolysis took up half of the shortest paragraph (lines 509 – 519) and an additional sentence (line 589 – 591), out of seven total paragraphs.

Specific comments:

(1) L137-138. Can *S. aureus* really survive for long periods of time outside of the host? Can you clarify this statement? Do you mean it is an opportunistic pathogen and can also replicate in the environment?

*S. aureus* can form biofilms and persist for weeks on inert surfaces (Kramer et al., 2024; Tran et al., 2023), indicating that it may replicate in non-host environments. We have included the phrase “opportunistic pathogen” to clarify (line 145).

(2) L187 - to ascertain

Corrected.

(3) Figure 2B - there seems to be a benefit of haemolysis activity to oxacillin resistance, perhaps a crossover in mechanism? In MSSA, without a host, it goes to complete fixation, whereas it is completely lost when antibiotics aren't present. I know this is discussed later, but I would appreciate a more detailed hypothesis of why this could be.

Antibiotics have been found to induce expression of virulence traits, such as in the case of oxacillin and hemolysis. Thus, it is reasonable that exposure to oxacillin during evolution would maintain MSSA’s hemolytic ability. We hypothesize that the loss of hemolysis in the absence of oxacillin may be due to the cost of hemolysis expression without a stimulant (oxacillin), hemolysis may not be expressed as often and be subject to deleterious mutations. Alternatively, the stress that cells were under favored virulence in some way, rather than the direct action of the antibiotic.

(4) L225-228 - As *C. elegans* do not have red blood cells, why would we expect this? Do you see increased lysis of *C. elegans* gut cells? Or could it be due to iron accumulation as you are growing the staph on BHI?

We measured and correlated nematode mortality with hemolytic ability because hemolysis had been found to be involved in virulence toward *C. elegans* (Sifri et al., 2003). The hemolysis phenotype is a surrogate for *S. aureus* virulence gene expression.

(5) Figure 3A - There seems to be a growth cost of evolving oxacillin resistance in the absence of a host. Why might this be?

MRSA populations exposed to oxacillin without a host during evolution visually exhibited the lowest growth rate. While this is an interesting question, the result was not statistically significant, so we cannot speculate in the manuscript.

**Reviewer #3 (Recommendations for the authors):**

(1) Some claims in the introduction are either non cited or not correctly stated. The second sentence has a claim about the interplay between antibiotic resistance and virulence with no citation listed. Additionally, there is a claim about *S. aureus* "evading detection" by attacking the host's immune cells. That is by definition not avoiding detection. Perhaps phrasing it as resisting host immune function would make it clearer.

We have added a citation (lines 80 – 81) and clarified our wording: “Once inside the host, *S. aureus* resists host immune function by hindering or lysing immune cells.” Lines 140 – 141

(2) Once in the introduction and in the discussion, the authors referred to *S. aureus* as a novel pathogen for *C. elegans*, I do not think enough is known to make this statement.

This *S. aureus* strain is novel because it was isolated from humans, so at least in its recent evolutionary past, it has not interacted with *C. elegans*. Furthermore, we used a *C. elegans* isolate (N2) that had been frozen and maintained in the lab on *E. coli*, and had not been exposed to other microbes in its recent evolutionary past. Finally, *S. aureus* has not been found to be a native pathogen of *C. elegans* in nature (Ekroth et al., 2021).

(3) Key suggestion: Change Figure 1C to reflect the design better. So you could have the +OXA before the host and then have an arrow looping back again to show the cycle of each step. So a figure that would have something like: MRSA > +OXA > +host>+OXA --> MRSA .

We have updated the figure as suggested.

(4) Suggest changing "greatest" on line 191, section header to greater.

Done.

(5) Line 258: Rich media can still provide selective pressures that are difficult to quantify - fast growth, cofactor and other nutrient limitations due to that fast growth

We have adjusted our wording: “Importantly, rich media reduced the risk of introducing additional selective pressures than those being tested.” Lines 273 – 274

(6) Why were intergenic mutations routinely ignored? These can often be very important phenotypically.

We had focused on genes because there was a sufficient number of genes to discuss, but we have added a Supplemental Table (Table S2) containing all mutations (including intergenic and synonymous) appearing in more than 2 populations. We have also added information regarding *mecA*, an accessory gene, highlighting the role non-core genes may have in shaping bacterial evolution:

“Despite evolving in similar environments, MRSA and MSSA populations differing only in the presence of an intact accessory gene (*mecA*)—proceeded on divergent evolutionary paths…” Lines 66 – 68

“Carriage of Staphylococcal cassette chromosome *mec* (*SCCmec*), which encodes *mecA*, an accessory gene that provides resistance…” Lines 187 – 188

“As MRSA and MSSA only differed in the presence of an intact *mecA* gene at the start of the experiment, accessory genes may play important roles in shaping bacterial evolution (Jackson et al., 2011).” Lines 472 – 474

(7) Line 294: more mutations than what?

We have clarified the sentence: “Sub-MIC oxacillin selection also resulted in more mutations than in its absence…” Lines 310 – 311

(8) Lines 295-297: wording is pretty confusing. It seems that the discussion is about increased mutation rates, possibly due to hypermutators resulting from mutL or recA mutations, but this isn't well-thought out and much is implied here. Furthermore, see the above comment about comparing mutations across conditions - it's hard to make inferences of mutation rates without knowing the mutation supply as a result of varying population sizes across conditions and through the experiment.

We have clarified the sentence: “…there were only two mutations in DNA and mismatch repair genes (*mutL* and *recA*), suggesting repair genes were not the sole mechanism involved.” Lines 313 – 314

Because all populations evolved from one ancestral clone (either MRSA or MSSA), all mutations that are found at the end of the experiment would have arisen de novo from that ancestor. Since all populations experienced the same number of passages/rounds of selection, we determined mutation rate by counting the number of mutations that were found at the last passage for each replicate population. Populations that acquired significantly more mutations had a higher mutation rate in terms of # of mutations/# of selection rounds.

(9) Line 486: typo "Mutations genes".

Corrected.

(10) Line 487: "antibiotics may allow" is awkward; suggest changing to more precise language, possibly relating to pleiotropy if that is what was meant here.

We had intended to mean “adaptation [to antibiotics] may allow”. We have clarified: “Mutations in genes involved in resistance to antibiotics were found more often in populations with increased virulence, suggesting that antibiotic adaptation may also favor evolution of virulence.” Lines 514 – 516

REFERENCES

Ekroth AKE, Gerth M, Stevens EJ, Ford SA, King KC. 2021. Host genotype and genetic diversity shape the evolution of a novel bacterial infection. ISME Journal 15:2146–2157. DOI: https://doi.org/10.1038/s41396-021-00911-3, PMID: 33603148

Kramer A, Lexow F, Bludau A, Köster AM, Misailovski M, Seifert U, Eggers M, Rutala W, Dancer SJ, Scheithauer S. 2024. How long do bacteria, fungi, protozoa, and viruses retain their replication capacity on inanimate surfaces? A systematic review examining environmental resilience versus healthcare-associated infection risk by “fomite-borne risk assessment.” Clinical Microbiology Reviews. PMID: 39388143

Sifri CD, Begun J, Ausubel FM, Calderwood SB. 2003. *Caenorhabditis elegans* as a model host for *Staphylococcus aureus* pathogenesis. Infection and Immunity 71:2208–2217. DOI: https://doi.org/10.1128/IAI.71.4.2208-2217.2003, PMID: 12654843

Tran NN, Morrisette T, Jorgensen SCJ, Orench-Benvenutti JM, Kebriaei R. 2023. Current therapies and challenges for the treatment of *Staphylococcus aureus* biofilm-related infections. Pharmacotherapy 43:816–832. DOI: https://doi.org/10.1002/phar.2806, PMID: 37133439
